# Artificial Intelligence and Nursing Management: Opportunities, Challenges, and Ethical Considerations—A Scoping Review

**DOI:** 10.1155/jonm/2797535

**Published:** 2025-07-30

**Authors:** Maryam Katebi, Masoud Bahreini, Razieh Bagherzadeh, Shahnaz Pouladi

**Affiliations:** ^1^Department of Nursing, School of Nursing and Midwifery, Bushehr University of Medical Sciences, Bushehr, Bushehr Province, Iran; ^2^Department of Midwifery, School of Nursing and Midwifery, Bushehr University of Medical Sciences, Bushehr, Bushehr Province, Iran

**Keywords:** artificial intelligence, decision making, ethical challenges, healthcare innovation, nursing management, scoping review

## Abstract

**Background:** The increasing complexity of healthcare necessitates exploring emerging technologies to enhance nursing management. Artificial Intelligence (AI) has shown significant potential in optimizing decision making, improving workflow efficiency, and enhancing resource allocation within nursing leadership. However, its implementation presents both opportunities and challenges, requiring a thorough understanding of its impact on managerial processes.

**Objective:** This scoping review aims to map the existing literature on AI applications in nursing management, identifying key benefits, limitations, ethical considerations, and future directions for AI integration in nursing management.

**Methods:** A scoping review methodology was employed, following the framework of Arksey and O'Malley (2005), refined by Levac et al. (2010), and adhering to the PRISMA-ScR guidelines. A systematic search of English and Persian databases, including PubMed, Web of Science, ProQuest, ScienceDirect, SID, and IranDoc, was conducted for studies published between 2013 and 2024. Thematic analysis was used to categorize findings across major domains of AI in nursing management.

**Results:** Twelve studies were included, featuring diverse methodologies and geographic locations. The key themes identified encompass the applications of AI in nursing management, where AI is utilized for data-driven decision making, workflow automation, staffing optimization, and patient monitoring. Challenges in AI implementation—Key concerns include data privacy, algorithmic bias, staff resistance, and limitations in interoperability. Potential benefits—AI contributes to greater efficiency, alleviates workload, and enhances predictive analytics for patient care. Ethical and practical considerations—There is a necessity for strong regulatory frameworks, training programs, and strategies to ensure the responsible integration of AI.

**Conclusion:** AI presents promising opportunities for nursing management, but its implementation requires careful consideration, including comprehensive training, ethical oversight, and organizational adaptation. Future research should investigate long-term implications, managerial intelligence, and workforce dynamics to enhance AI integration in nursing leadership.

## 1. Introduction

### 1.1. The Evolving Landscape of Nursing Management

Nursing management plays a pivotal role in ensuring efficient healthcare delivery, optimizing resource allocation, and maintaining patient safety in increasingly complex healthcare environments [1]. Contemporary healthcare systems face mounting pressures due to rising patient demands, evolving treatment modalities, and technological advancements. Traditional management strategies, such as performance-based payment systems, have been studied as potential methods to improve job satisfaction and care quality. However, findings indicate that financial incentives alone may not be sufficient to ensure sustained engagement and optimal performance [2]. Additionally, systemic thinking and emotional intelligence are crucial in enhancing managerial effectiveness, improving strategic decision making, and optimizing resource planning. Research highlights that leaders who adopt holistic, data-driven approaches tend to achieve better outcomes, yet traditional frameworks still depend heavily on human cognitive processing, which may introduce inefficiencies [2].

### 1.2. Artificial Intelligence (AI) in Healthcare: A Transformative Technology

AI has emerged as a revolutionary tool across various healthcare domains, offering capabilities such as data-driven diagnostics, predictive analytics, and automated administrative functions [3]. While AI has been widely adopted in clinical settings, its application in nursing management remains an evolving and underexplored area. Studies indicate that self-management models have demonstrated improvements in self-efficacy and coherence, allowing individuals to make informed healthcare decisions [4]. These findings reinforce the importance of technology-driven support systems in optimizing nursing administration. Moreover, research on pay-for-performance models suggests that awareness of compensation structures may enhance nursing care quality, but satisfaction levels remain moderate, pointing to the influence of intrinsic motivation and professional ethics [5]. This further supports the argument that AI-driven systems could help address managerial inefficiencies by incorporating automated decision support tools while considering ethical and motivational dynamics [1].

### 1.3. Scope and Rationale of the Review

Given the increasing presence of AI in healthcare, there is a critical need to map the current landscape of AI applications in nursing management and systematically identify the opportunities and limitations associated with its implementation. A scoping review approach is particularly suitable for this investigation, as it allows for a broad exploration of existing research without the constraints of a formal meta-analysis [2]. Unlike systematic reviews, which assess the effectiveness of interventions, scoping reviews aim to identify key concepts, thematic trends, and gaps in the literature [3].

This review seeks to

- Examine the range of AI applications currently utilized in nursing management.
- Identify the advantages and challenges associated with integrating AI into managerial processes.
- Highlight gaps in existing research and propose directions for future studies.

Through this broad investigation, this scoping review aims to provide insights into how AI is shaping nursing management, inform policy recommendations, and guide future research efforts aimed at optimizing AI's role within nursing leadership [6].

## 2. Methods

### 2.1. Study Design

This study was conducted as a scoping review, following the methodological framework outlined by Arksey and O'Malley and further refined by Levac et al. [7, 8]. Due to the exploratory nature of the research question, a scoping review was selected over a systematic review to map existing literature, identify key concepts, and outline gaps in knowledge regarding AI in nursing management. The review protocol was developed in accordance with the Preferred Reporting Items for Systematic Reviews and Meta-Analyses extension for Scoping Reviews (PRISMA-ScR) guidelines.

Scoping reviews are particularly valuable for examining the breadth of research on emerging topics without imposing strict inclusion criteria related to study design or methodology. The Arksey and O'Malley framework provides a structured approach, emphasizing iterative database searching, study selection, data extraction, and synthesis to comprehensively assess research trends [7]. Levac et al. expanded upon this methodology by refining criteria for study selection, emphasizing the need for analytical frameworks, and advocating for stakeholder engagement in scoping studies [8].

### 2.2. Search Strategy

A comprehensive literature search was conducted across multiple electronic databases to ensure inclusivity and depth in identifying relevant studies. The following databases were searched.

English databases: PubMed, Web of Science, Google Scholar, ProQuest, and ScienceDirect.

Persian databases: SID, Magiran, and IranDoc.

Notably, EMBASE and the Cochrane Library were omitted due to their primary focus on experimental studies, which were not central to the scope of this review. The search period was limited to 2013–2024 to reflect recent advancements in AI applications within nursing management. The search utilized a combination of Mesh terms and free-text keywords, including “nurse,” “nurse administrator,” “nurse manager,” “nursing management,” “artificial intelligence,” “computational intelligence,” “machine intelligence,” “AI,” “challenges,” “opportunities,” and “risks.” Boolean operators and truncation were applied to optimize search sensitivity.

### 2.3. Eligibility Criteria

Studies were included if they met the following criteria.

Published in English or Persian.

Full-text articles were available.

Explicitly mentioned relevant keywords in the title, abstract, or full text.

Addressed the application of AI in nursing management.

Studies were excluded if they focused on AI in broader healthcare disciplines (e.g., medical imaging and AI in diagnostics) without addressing managerial aspects.

### 2.4. Screening and Inclusion Strategy

Initially, a comprehensive database search yielded 1593 articles, which were imported into EndNote for reference management and deduplication. After removing duplicates, the studies underwent title and abstract screening to assess their relevance to AI applications in nursing management. Unlike systematic reviews, which often exclude studies based on methodological quality, this scoping review prioritized inclusivity by incorporating a wide range of study designs to capture the breadth and diversity of the literature on the subject.

The full-text review was conducted independently by two reviewers who evaluated articles based on conceptual relevance rather than predefined methodological rigor. Disagreements were resolved through discussion, ensuring that the studies aligned with the research objectives while maximizing the scope of the review.

### 2.5. Framework for Study Selection

The selection process followed five key stages, as recommended by Levac et al. [8].

Identifying relevant studies—Based on predefined keywords and database searches.

Screening titles and abstracts—Removing irrelevant studies while retaining those addressing AI in nursing management.

Full-text review—Assessing depth of discussion related to AI integration in nursing leadership and managerial decision making.

Charting key data—Extracting information relevant to AI applications, challenges, opportunities, and managerial impacts.

Collating and summarizing results—Synthesizing findings into thematic categories for further analysis and interpretation.

Unlike systematic reviews, where risk of bias assessments may be crucial for evidence synthesis, this scoping review did not perform a formal bias evaluation. Instead, studies were mapped thematically to explore trends, knowledge gaps, and emerging perspectives on AI in nursing management.

### 2.6. Data Extraction

Two reviewers independently extracted data using a structured extraction form, summarizing:

• Study title, authorship, year, country, and journal
• Study design, sample size, and methodology
• AI application, managerial relevance, and outcomes
• Ethical considerations and implementation challenges
• Overall study quality using MMAT (2018)

Missing data were addressed through author contact where feasible, or expert consultation in cases of nonresponse.

### 2.7. Data Synthesis

A descriptive approach was employed, categorizing AI applications in nursing management based on thematic content analysis. Due to heterogeneity across study designs, no meta-analysis was conducted. A risk of bias assessment was not formally performed, as the scoping review aimed to map the evidence rather than evaluate the effectiveness of interventions.

### 2.8. Ethical Considerations

Since this study did not involve human participants, institutional ethical approval was not required. Transparency in reporting and adherence to academic integrity principles were ensured.

## 3. Results

### 3.1. Overview of Included Studies

This scoping review identified 12 studies that explored the application of AI in nursing management. The selected studies originated from various geographic regions, particularly in Europe, Asia, and North America. The included studies employed a diverse range of methodologies, such as qualitative analysis, descriptive approaches, exploratory methods, and mixed-method designs, reflecting the breadth of research on this emerging topic.

Unlike systematic reviews that evaluate the methodological rigor of selected studies, this scoping review mapped existing knowledge by identifying key themes related to AI applications, challenges, and future implications for nursing management. Studies were categorized based on their thematic relevance rather than their compliance with specific quality appraisal metrics.

### 3.2. Thematic Analysis of AI in Nursing Management

The findings were organized into four major categories, as presented in Table 1, which synthesizes key trends from the existing literature.

### 3.3. Applications of AI in Nursing Management

The studies reviewed indicated that AI is increasingly applied in nursing administration, particularly in workflow optimization, data-driven decision making, resource management, and automation of routine tasks. AI-driven tools have been found to enhance hospital staffing efficiency and enable predictive analytics for patient monitoring. These applications enable nurse managers to make informed decisions, ultimately enhancing patient care and operational efficiency.

### 3.4. Challenges in AI Implementation

Despite the potential advantages of AI, several studies highlighted critical challenges in its implementation. Ethical concerns, such as data privacy, algorithmic bias, and patient confidentiality, remain significant barriers to widespread adoption. Furthermore, resistance from nursing staff, stemming from fears of job displacement and insufficient training programs for AI literacy, is often cited as an obstacle. Interoperability issues between AI-based systems and existing healthcare infrastructures also emerged as a recurring theme.

### 3.5. Potential Benefits of AI in Nursing

The review found that AI can help reduce nursing workloads, enhance patient care decision making, and improve hospital resource management. AI-driven automation of administrative tasks allows nurse managers to shift their focus toward patient-centered care. Several studies suggested that AI-powered tools can increase efficiency in triage systems and support evidence-based decision making, resulting in more strategic workforce allocation.

### 3.6. Ethical and Practical Considerations

Given AI's increasing role in healthcare, regulatory and ethical frameworks must be established to ensure its responsible integration into nursing management. Transparent AI decision-making processes, stakeholder involvement, and interdisciplinary collaboration will be essential for reducing the risks associated with AI adoption in nursing. Studies highlight the significance of maintaining human-centered nursing practices, ensuring that AI serves as a supportive tool rather than a replacement for human expertise.

## 4. Discussion

This scoping review provides a broad exploration of the existing literature on AI in nursing management, highlighting key applications, challenges, opportunities, and ethical considerations. Unlike systematic reviews that focus on synthesizing findings through rigorous quality appraisal, this study aimed to map available evidence and identify gaps requiring further exploration [6, 7].

### 4.1. The Evolving Role of AI in Nursing Management

Findings suggest that AI is increasingly integrated into nursing management, specifically in resource allocation, workflow optimization, predictive analytics, and clinical decision support [6, 9]. AI-powered systems assist in reducing administrative burdens, allowing nurse managers to dedicate more time to patient-centered care. However, despite its potential, AI adoption remains uneven across healthcare settings due to technological limitations, resistance from nursing staff, and infrastructure concerns [13].

Recent studies have emphasized AI's role in clinical decision support systems, patient monitoring, and nursing education. AI-driven predictive models have been shown to enhance early detection of patient deterioration, improving response times and overall patient outcomes [15]. However, the literature also highlights significant gaps, particularly in the development of nursing-specific AI tools tailored to the unique demands of nursing workflows [14].

### 4.2. Challenges in Implementation

Several studies emphasized barriers to AI adoption in nursing, including ethical concerns regarding data privacy, algorithmic bias, and the potential loss of human-centric decision making [12]. Many nurse managers expressed apprehension about AI's role in replacing traditional nursing responsibilities, underscoring the need for clear guidelines on AI integration to preserve nursing autonomy and ethical integrity [19].

Additionally, staff training and AI literacy emerged as critical factors influencing the success of implementation—without adequate education, AI tools may fail to deliver expected benefits [11]. A comparative analysis of AI-driven nursing care plans found that AI models could generate efficient and accurate care plans significantly faster than human nurses, yet professionals expressed dissatisfaction with current diagnostic documentation systems, highlighting the need for human oversight and ethical considerations [20].

### 4.3. Potential Benefits and Knowledge Gaps

AI offers notable advantages, including improved efficiency, enhanced managerial decision making, and optimized patient safety measures [10]. AI-driven automation of administrative tasks allows nurse managers to redirect their focus toward patient-centered care. Several studies suggested that AI-powered tools can increase efficiency in triage systems and facilitate evidence-based decision making, leading to more strategic workforce allocation [15].

Despite these promising developments, research on the long-term consequences of AI integration in nursing management remains limited [17]. The role of managerial intelligence in AI-driven decision making is an area requiring deeper investigation. Additionally, geographic disparities in AI adoption, particularly in regions with lower technological infrastructure, highlight the need for global strategies to ensure equitable access to AI-driven nursing management solutions [19].

### 4.4. Ethical and Practical Considerations

The review underscores the necessity for robust ethical frameworks governing AI use in nursing [18]. As AI-driven algorithms handle increasingly complex decisions, concerns regarding transparency, fairness, and accountability become more pronounced [12]. Regulatory bodies must establish clear policies on data security, AI bias mitigation, and job displacement to ensure ethical deployment of AI technologies in nursing management. Nurse managers must also remain actively engaged in evaluating AI tools to ensure alignment with core nursing values.

## 5. Conclusion

This scoping review highlights the current state of AI in nursing management, identifying significant applications, challenges, and ethical concerns. AI holds transformative potential in optimizing managerial processes; however, successful implementation depends on staff readiness, technological infrastructure, ethical regulations, and interdisciplinary collaboration. To support the responsible adoption of AI, healthcare organizations must invest in comprehensive training programs that ensure nurse managers acquire AI literacy while preserving a human-centered approach to care. Additionally, researchers should further explore the long-term impact of AI on managerial decision making, nursing autonomy, and workforce dynamics.

Future studies should move beyond descriptive analyses to investigate the real-world integration of AI, focusing on its effects on nursing workflow efficiency, patient safety, and its ethical implications. By addressing these gaps, nursing leadership can harness AI's strengths while safeguarding the integrity and autonomy of the nursing profession.

## Figures and Tables

**Table 1:** Thematic categorization of artificial intelligence applications in nursing management: a scoping review.

Category	Key findings	Representative studies
Applications of AI in nursing management	AI is used for workflow optimization, data-driven decision making, automated patient monitoring, and resource allocation.	Chang et al. [9] Vandresen et al. [10] Seibert et al. [11]

Challenges in AI implementation	Ethical concerns (data privacy and algorithmic bias), resistance from nursing staff, inadequate training, and interoperability issues between AI systems.	Petersson et al. [12] Laukka et al. [13] Elsayed and Sleem [14]

Potential benefits of AI in nursing	Improved efficiency, reduced nursing workload, enhanced predictive analytics for patient care, and better resource management.	Huang et al. [15] Abd Elhamid et al. [16] Buchanan et al. [17]

Ethical and practical considerations	There is a need for regulatory frameworks, transparency in AI decision making, and the maintenance of human-centric nursing practices.	Ergin et al. [18] Fernandes et al. [19] Stahl et al. [20]

## Data Availability

This scoping review is based exclusively on previously published literature and publicly accessible resources, all of which have been appropriately identified and cited within the manuscript. As no primary data were collected or generated during the course of this study, data sharing is not applicable.
